# Redox Signaling Pathways Involved in Neuronal Ischemic Preconditioning

**DOI:** 10.2174/157015912804143577

**Published:** 2012-12

**Authors:** John W Thompson, Srinivasan V Narayanan, Miguel A Perez-Pinzon

**Affiliations:** Cerebral Vascular Disease Research Center, Department of Neurology, University of Miami, Miller School of Medicine, Miami, Fl 33136

**Keywords:** Epsilon PKC, HIF, Ischemic preconditioning, Nrf2, Reactive oxygen species, Sirtuin, SIRT1, Reperfusion.

## Abstract

There is extensive evidence that the restoration of blood flow following cerebral ischemia contributes greatly to the pathophysiology of ischemia mediated brain injury. The initiating stimulus of reperfusion injury is believed to be the excessive production of reactive oxygen (ROS) and nitrogen (RNS) species by the mitochondria. ROS and RNS generation leads to mitochondrial protein, lipid and DNA oxidation which impedes normal mitochondrial physiology and initiates cellular death pathways. However not all ROS and RNS production is detrimental. It has been demonstrated that low levels of ROS production are protective and may serve as a trigger for activation of ischemic preconditioning. Ischemic preconditioning is a neuroprotective mechanism which is activated upon a brief sublethal ischemic exposure and is sufficient to provide protection against a subsequent lethal ischemic insult. Numerous proteins and signaling pathways have been implicated in the ischemic preconditioning neuroprotective response. In this review we examine the origin and mechanisms of ROS and RNS production following ischemic/reperfusion and the role of free radicals in modulating proteins associated with ischemic preconditioning neuroprotection.

## INTRODUCTION

The high rate of energy consumption by the brain renders it extremely sensitive to brief periods of oxygen and/or glucose deprivation, such as occurs during anoxia, ischemia and hypoglycemia. Oxygen deprivation in the brain leads to a decline in cellular ATP levels, resulting in an inability of the brain to maintain ion gradients. Failure to maintain ionic gradients can result in uncontrolled cation influx, depolarization, and indiscriminate release of the excitatory neurotransmitter glutamate. The ensuing glutamate excitotoxicity increases cytoplasmic calcium levels, resulting in mitochondrial dysfunction and cell death. 

Ischemia hinders mitochondrial function affecting its maintenance of ATP levels and contributing to free radical production. This mitochondrial dysfunction has received considerable attention as a major contributor to both ischemia and reperfusion mediated injury [1-3]. Numerous studies have demonstrated that mitochondrial respiration is severely affected by ischemia and by reperfusion [4-9]. Hyperoxidation of respiratory chain proteins is characteristic of the mitochondria following ischemia and may stem from substrate unavailability, production of reactive oxygen species (ROS), or through the release of cytochrome c and NADH following mitochondrial permeability transition pore opening (MPTP) [10, 11]. Mitochondrial dysfunction following ischemia is the primary site of free radical production and oxidative damage in the reperfused brain following ischemia [1, 12-14]. Previous studies have extensively demonstrated that tissue reperfusion following an ischemic episode contributes significantly to the pathophysiology of ischemic injury [15-19]. 

## ISCHEMIC PRECONDITIONING

Ischemic preconditioning (IPC) is an intrinsic neuro-protective mechanism whereby a brief, sublethal ischemic insult protects against a subsequent lethal ischemic attack. IPC has been demonstrated in numerous organisms and tissues by many groups. IPC is characterized by an early window of tolerance which occurs within minutes following the sublethal ischemic insult and lasts a few hours [5, 20]. This early window of protection is followed approximately 24 - 48 hours later by a second window of protection, which persists for days to weeks. 

There are many trigger mechanisms which are implicated in initiating the preconditioning response, including neuroactive cytokines [21, 22], adenosine receptors [23] [20], ATP-sensitive potassium channels [24-28], and oxidative stress [29]. During early reperfusion following IPC, the neuroprotective response is quickly initiated through the posttranslational modification of proteins, whereas the second or delayed window of protection is characterized by alteration in gene expression. One aspect of IPC mediated protection is the regulation of excessive ROS formation following ischemia/ reperfusion. 

## REACTIVE OXYGEN SPECIES

The generation of ROS occurs under normal physiological conditions and is normally balanced by the cellular antioxidant defense system. However, ROS formation may be increased during periods of ischemia and reperfusion. Previous studies have suggested that the increased rate of ROS production during reperfusion is a major cause of the pathophysiology of ischemia/reperfusion injury in both the heart and brain [1, 12 , 30, 13, 31].

**Fig. (1):**
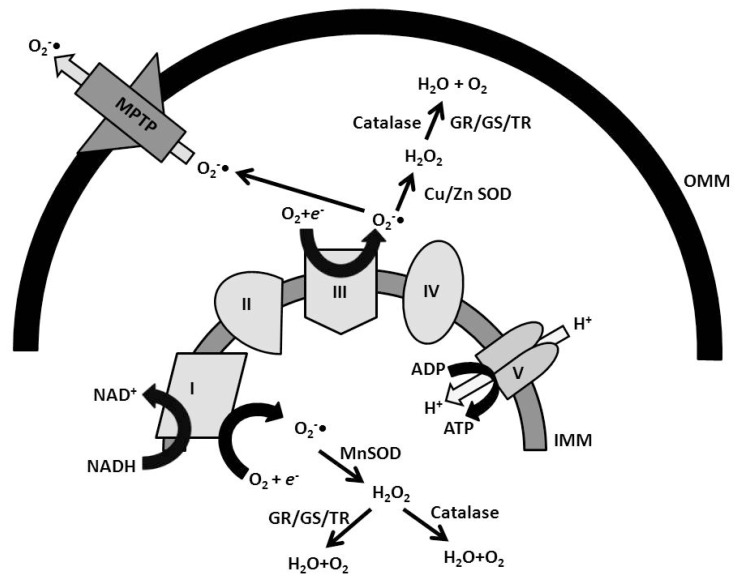
**Summary diagram of ROS production and ROS elimination in the mitochondria**. ROS is endogenously produced primarily from complex I and III of the ETC in the mitochondria. The production of ROS would quickly buildup if there were not mechanisms in places to quickly convert ROS into inert byproducts. In the matrix of the mitochondria, O_2_^-•^ is formed between molecular oxygen reacting with electrons from the ETC. In the case of ischemia, oxygen is limiting and thus transport of electrons through the ETC stalls; stalling of the ETC allows more time for electrons to react with remaining intramitochondrial oxygen and form the potentially devastating O_2_^-•^ ion. However, the matrix of the mitochondria contains MnSOD, which converts O_2_^-•^ into H_2_O_2_. The hydrogen peroxide can be further reduced to water and oxygen through catalase or glutathione/thioreduxin reduction pathways, both of which are conveniently located in the mitochondrial matrix. ROS can also be generated in the intermembrane space of mitochondria from complex III. In the intermembrane space, O_2_^-•^ is acted upon by either ZnSOD or CuSOD, and reduced to H_2_O_2_. However, if O_2_^-•^ exceeds the capacity of these enzymes, O_2_^-•^ could activate opening of the MPTP, releasing *cyt*C and stimulating apoptotic pathways. To prevent this occurrence, ROS plays a role in cell signaling, and stimulates transcription of cytoprotective genes involved in adaptation to oxidative stress and antioxidant expression. Together, the induction of these cytoprotective genes will eventually reduce ROS formation; this negative feedback provides the continual generation of enzymes designed to quell a significant rise in ROS production before irreversible cell damage and cell death occur. MnSOD: Manganese Super Oxide Dismutase; CuSOD: Copper Super Oxide Dismutase; ZnSOD: Zinc Super Oxide Dismutase; O_2_^-•^: Superoxide ion; mtDNA: mitochondrial DNA; *cyt*C: cytochrome C; MPTP: Mitochondrial Permeability Transition Pore; O_2_ : molecular oxygen; *e*^-^: electron; ETC: Electron transport chain; I-V: Denotes complex number of ETC; ADP: Adenosine diphosphate; ATP: Adenosine triphosphate; IMM: Inner mitochondrial membrane; OMM: Outer mitochondrial membrane; NADH: Nicotinamide adenine dinucleotide; GR: Gluthathione reductase; GS: Glutathione synthase; TR: Thioreduxin ; H_2_0: water; O_2_: molecular oxygen.

Multiple sites of ROS production have been suggested, with the mitochondria accounting for the majority of the ROS generated inside cells (Fig. **1**). ROS are produced primarily by complex I and III of the mitochondrial electron transport chain [32]. In organotypic hippocampal slice cultures, ischemia/reperfusion-mediated free radical formation was reduced by the complex I inhibitor rotenone [33]. During conditions of hypoxia, the electron transport chain stalls, allowing donated electrons from NADH^+^ and FADH_2 _sufficient time to interact with oxygen in the mitochondria. The main types of ROS produced by the mitochondria are superoxide and hydrogen peroxide. Hydrogen peroxide, if not degraded by antioxidants, will form highly reactive hydroxyl radicals, which increase during the early reperfusion phase following brief periods of global cerebral ischemia [34]. Increases in oxidative stress, occurring either by increased ROS formation or decreased antioxidant defenses, result in lipid peroxidation, and protein and DNA damage (Table **1**). 

## THE ROLE OF ROS IN IPC NEUROPROTECTION

In contrast to excessive ROS generated by ischemia/reperfusion, there is evidence that mild amounts of ROS production are protective and may serve as a trigger for activating IPC protective pathways [35-39]. For example, perfusing the isolated heart with the antioxidant N-acetylcysteine abolished IPC induced cardioprotection [40]. Similarly in the brain, inhibition of superoxide dismutase (SOD) induced ROS production and neuroprotection against ischemia [29]. The importance of ROS in IPC neuro-protective signaling is further demonstrated from studies showing that the use of ROS scavengers such as SOD and catalase decreased IPC-mediated protection in canine hearts [41]. In addition, a study in rats suggested that ROS signaling mediates IPC-induced neuroprotection [42]. In this study by Puisieux *et al., *pretreatment with the free radical scavenger dimethylthiourea (DMTU) or the antioxidant ebselen increased infarct volume in IPC-treated rats following middle cerebral artery occlusion [42]. The data suggested that an initial oxidative stress may initiate the protective pathways of IPC. Another study by Liu *et al.,* yielded comparable results; utilizing transgenic mice overexpressing human superoxide dismutase (SOD1), wild type and transgenic embryonic mouse cortical neurons were cultured and subjected to IPC (2 hours of anoxia) and then severe anoxia (15 hours anoxia). IPC was shown to be protective in wildtype mouse neuronal cultures, but this protection was significantly decreased in transgenic cultures, further suggesting the importance of ROS in triggering IPC protective pathways [43].

The mechanism by which ROS is generated during the initial phase of IPC appears to be related to the opening of the mitochondrial ATP sensitive potassium channels (mitoK^+^_ATP_). In both the brain [44] and heart [45] opening of the mitoK^+^_ATP_ channels occurs early in the preconditioning response and is required for IPC protection. The use of a mitoK^+^_ATP_ channel antagonist, such as 5-hydroxydecanoic acid, blocked IPC-mediated protection in the rat heart [46] whereas the mitoK^+^_ATP_ channel agonist, diazoxide, induced a preconditioning response [38, 47, 48]. The opening of the mitoK^+^_ATP_ channel has been suggested to lead to generation of ROS. In the heart, the protective effect of diazoxide was blocked in the presence of antioxidants. These results implicate a requirement of mitoK^+^_ATP_ in ROS formation [47, 48]. In the rat hippocampal slices, opening of the mitoK^+^_ATP_ channel with diazoxide protected against oxygen and glucose deprivation induced cell death which could be prevented by the ROS scavenger N-2-mercaptopropionyl glycine [49]. Thus, there exists a delicate balance in ROS formation such that high levels of ROS generated during ischemia/reperfusion is cytotoxic, whereas low levels of ROS generated by IPC is neuroprotective.

## NITRIC OXIDE

**Fig. (2):**
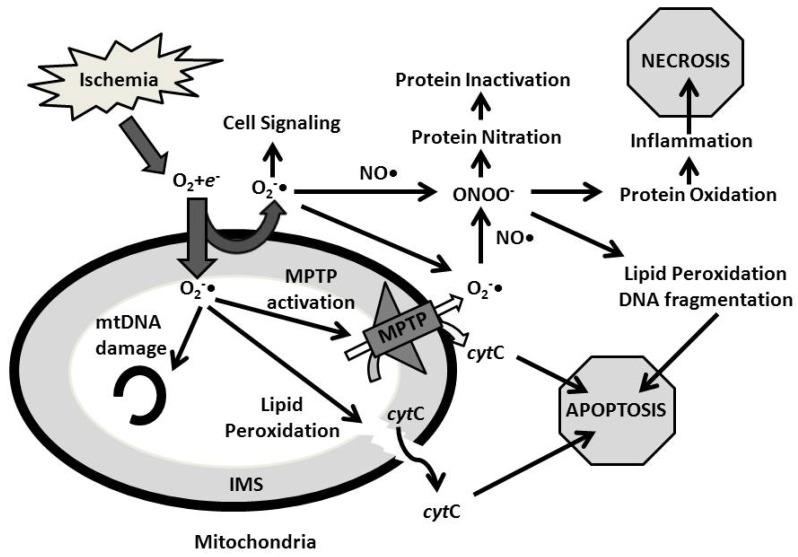
**Summary diagram of cytotoxic effects resulting from ROS and RNS generation**. During ischemia, ROS is produced from complexes I and III of the ETC in the mitochondria. ROS can activate several pathways leading to cell damage and cell death. O_2_^-•^ can oxidize and fragment both mtDNA and nuclear DNA of a cell. mtDNA damage results in decreased synthesis of ETC proteins, resulting in decreased ATP production. In addition, DNA damage can activate DNA repair enzymes, and if damage exceeds the capacity of these repair enzymes, the cell will be signaled for apoptotic cell death. ROS produced in the mitochondria can also oxidize mitochondrial membranes, eventually leading to leakage of *cyt*C, a pro-apoptotic signaling molecule. Similarly, ROS can stimulate opening of the MPTP, leading to loss of mitochondrial membrane potential and leakage of *cyt*C from the intermembrane space, both of which contribute to activation of apoptosis. If O_2_^-•^ were to react with NO residing in close proximity, ONOO^-^ is formed. ONOO^-^ has a multitude of effects, sharing several downstream pathways with ROS. ONOO^-^ can nitrate, and thus inactivate, many proteins. Some targets include SOD, DNA repair enzymes, and myelin. The result is increased burden of oxidative stress and inability to cope with ROS, especially in the nervous system. In addition, ONOO^-^ can oxidize proteins, such as LDL, in vascular tissue. This process stimulates potent inflammation and compromises vascular integrity. Similar to ROS, ONOO^-^ can oxidize membrane lipids and fragment DNA, all of which contribute to apoptosis. However, low levels of ROS which do not exceed the coping ability of the cell may be potentially beneficial, as these radical species may activate several cytoprotective and adaptive pathways to ameliorate oxidative stress. ONOO^-^ : Peroxynitrate; O_2_^-•^: Superoxide ion; mtDNA: mitochondrial DNA; *cyt*C: cytochrome C; MPTP: Mitochondrial Permeability Transition Pore; O_2_ : molecular oxygen; *e*^-^: electron; ETC: Electron transport chain; IMS: Intermembrane space.

Another reactive species implicated in post-ischemic cell damage is nitric oxide (NO) [50]. NO is a free radical gaseous molecule that regulates several physiologic processes. NO may react with other oxygen species such as O_2_ and hydrogen peroxide to generate radical nitrogen species, such as peroxynitrite (ONOO^−^) (Fig. **2**). NO can directly regulate certain proteins through S-nitrosylation. There are three isoforms of nitric oxide synthase (NOS): endothelial NOS (eNOS), inducible NOS (iNOS), and neuronal NOS (nNOS). Mice overexpressing eNOS had reduced infarct size when compared to wild type littermates following cardiac ischemia [51], suggesting that NO plays an important role in protective signaling following oxidative stress. NO activates guanylate cyclase, which stimulates the production of cGMP and subsequent activation of protein kinase G (PKG). Nitric oxide has also been shown to affect mitochondrial function, and mediate protection through various mechanisms. A previous study suggested nitric oxide mediated opening of the mitoK^+^_ATP_ [52], NO was also proposed to inactivate the electron transport chain by inhibiting electron entry into the electron transport chain, and also aiding in the generation of low levels of ROS [53]. NO can also mediate a dampening response following reperfusion by slowly reactivating electron transport chain functioning. This slow activation of the electron transport chain following reperfusion attenuates calcium overload, ROS generation, and MPTP activation [54, 55]. NO may also compete with oxygen to bind to and inhibit cytochrome *c* activity when oxygen is limiting, potentially activating ROS generation from upstream of the electron transport chain mitochondrial complexes [56]. NO can S-nitrosylate several targets involved in respiration and mitochondrial functioning, including cytochrome oxidase [57] and dynamin related protein 1 (DRP-1) [58], a protein associated with mitochondrial fission and autophagy. By stimulating autophagy, mitochondrial ROS production can be attenuated following exposure to severe hypoxic stress [59]. 

## REACTIVE NITROGEN SPECIES

In addition to ROS and NO, reactive nitrogen species (RNS) are another group of molecules with high oxidative capacities. These molecules are formed under normal physiologic conditions, and can have important implications in cell signaling, oxidative damage, and cell death following ischemia. Following inflammatory processes, cells of the immune system produce both superoxide and NO molecules, which react to form peroxynitrite. Similarly, NO produced by neurons may react with superoxide following cerebral ischemia. This pathologic process can favor the production of RNS over beneficial NO [60]. Peroxynitrite is the predominant form of RNS; dissociation reactions of peroxynitrite can also lead to the formation of nitrosonium cation (NO^+^) and the nitroxyl anion (NO^−^), which is implicated in similar signaling cascades and cell damage as peroxynitrite [60]. 

## CONTRIBUTION OF ROS/RNS TO ISCHEMIA MEDIATED CELL DEATH

Peroxynitrite is formed rapidly when superoxide and nitric oxide are produced in close proximity. Peroxynitrite can oxidize cysteine residues of various proteins, including Complex I, II, III, and V of the mitochondrial electron transport chain [61]. Upon cysteine oxidation by peroxynitrite, these protein targets are inactivated, and thus peroxynitrite can inhibit energy production by the cell. However, as peroxynitrite inhibits complex I and III in mitochondria, low concentrations of this RNS may also serve to reduce ROS generation. 

Tyrosine nitration is another chemical modulation mediated by peroxynitrite, and may be an *in vivo* marker to measure nitrosative stress. Peroxynitrite can cause tyrosine nitration of mitochondrial manganese superoxide dismutase (MnSOD), resulting in inactivation of this enzyme [62, 63]. Interestingly, inactivation of MnSOD results in impaired dismutation of the superoxide generated primarily by complex I and III in the mitochondria; therefore, the resulting increase in superoxide favors increased formation of peroxynitrite, effectively creating a positive feedback loop. Another protein targeted by peroxynitrite-mediated tyrosine nitration is prostacyclin (PGI_2_) synthase [64]. Inactivation of this enzyme results in a decrease in PGI_2 _synthesis, contributing to vascular pathology and inflammation in ischemic/reperfusion injury [65]. Additionally, α-synuclein can be nitrated at specific tyrosine residues, contributing to neurodegenerative diseases and accumulation of Lewy Body’s as occurs in Parkinson’s Disease [66]. Thus, the role of reactive oxygen and nitrogen species in neurodegenerative diseases has been well established by previous studies. 

Lipid oxidation by peroxynitrite is a cytotoxic event similarly caused by other free radical species such as ROS [67]. Peroxynitrite can initiate peroxidation of myelin in the central nervous system, and contribute to inflammatory cerebral disease and subsequent demyelination [68]. In addition, the potent oxidative capacity of peroxynitrite can result in oxidation of low-density lipoprotein (LDL), a critical event in vascular inflammation, plaque accumulation, and progression of arterial disease [69]. Finally, DNA fragmentation and apoptosis are also mediated by peroxynitrite [70]. Peroxynitrite, similar to superoxide and ROS, can create DNA strand breaks and poly (ADP-ribose) polymerase (PARP) activation, which activates a family of proteins involved in DNA repair and apoptosis [71]. Through PARP activation, DNA strand breaks can then induce apoptosis and cell death through subsequent caspase activation. In the presence of high peroxynitrite levels, the increased recruitment of PARP-1 depletes NAD^+ ^[72], because PARP-1 requires NAD^+^ to perform its DNA repair function. Lowered NAD^+^ levels result in decreased ATP production and subsequent necrotic cell death [72]. Thus, both apoptosis and necrosis can be seen from increased peroxynitrite concentration secondary to ischemia. 

## EFFECTS OF IPC ON ROS/RNS

The use of peroxynitrite scavengers has been shown to reduce the beneficial effects of IPC on arrhythmias in rat hearts. This study suggested that low concentrations of peroxynitrite are beneficial and may contribute to IPC mediated protection [73]. Another study suggested that IPC prevented a rise in peroxynitrite concentration following ischemia-reperfusion in rat hearts subjected to global ischemia for 30 minutes. However, preconditioning initially increased tyrosine nitration levels of proteins, thus suggesting that initially peroxynitrite may be increased, but is greatly attenuated following lethal ischemic insults [74].

## ENDOGENOUS ANTIOXIDANTS

The cell has several defense mechanisms against oxidative stress, such as antioxidant enzymes, that aid to quell a substantial rise in ROS levels following exposure to ischemia (Fig. **1**). Part of IPC protection stems from its ability to up-regulate the cellular antioxidant defense system. 

The super oxide ion, or O_2_^-^, is formed under normal physiologic conditions upon the reaction of molecular oxygen (O_2_) and an electron. The electron may be donated from complex I or complex III of the mitochondrial electron transport chain, or from other metabolic reactions in the cell. These other reactions include xanthine oxidase, alpha-ketoglutarate, cyclooxygenase and lipoxygenase pathways [75]. In the cytoplasm of the cell, copper/zinc (Cu/Zn) superoxide dismutase (SOD1) converts superoxide into hydrogen peroxide (H_2_O_2_). Similarly, manganese superoxide dismutase MnSOD2 in the mitochondria converts superoxide produced in the mitochondrial matrix into hydrogen peroxide as well [76]. 

Further removal of hydrogen peroxide is performed through the actions of the glutathione and thioredoxin reductase systems, both of which decompose hydrogen peroxide molecular oxygen and water [77]. Catalase is another enzyme that can reduce hydrogen peroxide, and is found in peroxisomes [78]. Lastly, NAD(P)H quinone oxidoreductase 1 (NQO1) may scavenge superoxide [79], as well as reducing endogenous quinones such as vitamin E quinine and coenzyme Q10 in the mitochondrial ETC [80]. The reduction of these molecules produces more stable quinones, which increase their antioxidant ability. Coenzyme Q10 may react with vitamin E to scavenge free radicals that accumulate in the mitochondria, and increased accumulation of coenzyme Q10 has been shown to be neuroprotective in various animal models of neurodegeneration [81, 82]. 

The regulation of most of these antioxidant enzymes occurs through the transcription factor nuclear factor erythoid-2 related factor (Nrf2) [83]. Nrf2 regulation of the antioxidant system will be reviewed in more depth later in this review, but in summary, Nrf2 dissociates from its cytosolic repressor protein following electrophilic and oxidative stress, and will translocate to the nucleus to transcribe endogenous antioxidant genes [84]. Thus, activation of Nrf2 is an important pathway that can upregulate endogenous antioxidant production, and prevent the pathogenesis of ischemic injury. 

### Hydrogen Sulfide 

Hydrogen sulfide (H2S) is a gaseous neurotransmitter which has been demonstrated to regulate numerous physiological processes including apoptosis, vascular tone, mitochondrial metabolism and antioxidant enzyme levels [85, 86]. H2S is synthesized from L-cysteine and homocysteine by the enzymes cystathionine β-synthase (CBS) and cystathionine γ-lyase (CSE) [87]. CBS is expressed primarily in the central nervous system whereas CSE is expressed primarily in the cardiovascular system [88, 89]. H2S defends against oxidative stress by several mechanisms [90, 91]. In primary cortical neuronal cultures H2S was demonstrated to increase cellular levels of the antioxidant, glutathione by maintaining cellular cystine levels, a precursor to glutathione, and by enhancing the activity of γ-glutamyl-cystine synthetase, a rate limiting enzyme in glutathione production [91]. In the heart, exogenous H2S therapy was protective against ischemia/reperfusion injury by increasing AKT phosphorylation and by increasing nuclear localization of two transcription factors, nuclear respiratory factor 1 and Nrf2 both of which are involved in regulating the expression of antioxidant enzymes [92]. The cardioprotective effects of H2S have also been demonstrated to act as a preconditioning mimetic. Calvert *et al*., [93] demonstrated that H2S administered 24hrs prior to myocardial ischemia increased nuclear localization of Nrf2 and increased phosphorylation of PKC epsilon, a key regulatory protein in the preconditioning response. It was also observed that H2S preconditioning upregulated the expression of heme oxygenase-1 and thioredoxin-1, two key antioxidants, during late preconditioning. 

### Carbon Monoxide

A second gaseous neurotransmitter associated with oxidative stress is carbon monoxide (CO). CO is produced during heme metabolism by the enzyme, heme oxygenase [94]. There are two forms of heme oxygenase expressed in the brain. Heme oxygenase-2 is constitutively expressed while heme oxygnease-1 is expressed during periods of oxidative stress such as occurs during ischemia/reperfusion [95-98]. CO has been demonstrated to regulate apoptosis and inflammation primarily thru activation of mitogen-activated protein kinase (MAPK) signaling protein p38. The antioxidative properties of CO are primarily regulated thru the Nrf2 transcription factor pathway. Wang *et al*., [99] demonstrated that CO administration 18hrs following permanent middle cerebral artery occlusion increased nuclear localization of Nrf2 and heme oxygnease-1 expression, and was associated with reduced infarct volume and neurological deficits. They also demonstrated that the neuroprotective effects of CO were abolished in Nrf2-knockout mice, suggesting a vital role for Nrf2 in CO mediated neuroprotection. 

CO has also been demonstrated to generate low levels of mitochondrial ROS formation under normal physiological oxygen levels [100, 101]. In highly metabolically active tissues, such as the brain and heart, CO has been demonstrated to bind to cytochrome oxidase which produces reduction responses in cytochrome bc1 portion of the electron transport chain, as well as, ROS generation [102]. However, the physiological consequences of CO induced mitochondrial ROS production are not fully understood. 

## MECHANISMS OF IPC INDUCED ROS AND RNS PROTECTION

**Fig. (3):**
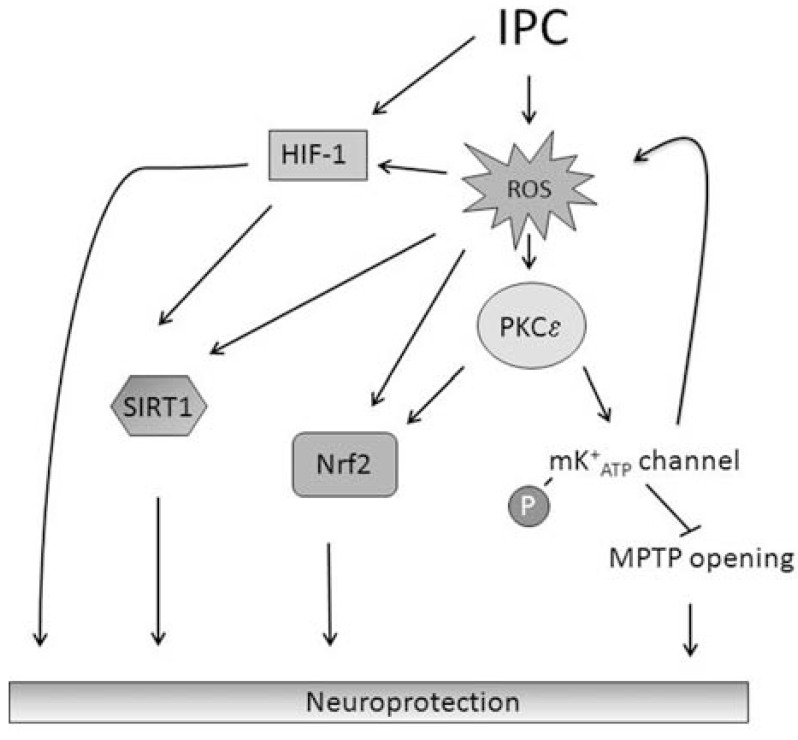
**Neuroprotective signaling pathways activated by ROS following ischemic preconditioning (IPC)**. Low levels of ROS formation following IPC exposure have been implicated in activating numerous signaling pathways involved in IPC neuroprotection. Of the proteins targeted by ROS, PKCε is central to IPC mediated neuroprotection. Once activated, PKCε phosphorylates and thereby opens the mitoK^+^_ATP_ channel leading to further ROS formation by the mitochondria and inhibiting the opening of the mitochondrial permeability transition pore (MPTP). Low levels of ROS are also known to activate HIF-1, SIRT1, and Nrf2. ROS may also regulate neuroprotective signaling pathways indirectly through signaling pathway cross-talk. ROS: Reactive oxygen species; IPC: Ischemic Preconditioning; PKCε: Protein Kinase C Epsilon; mitoK^+^_ATP_: mitochondrial ATP sensitive potassium channel; MPTP: Mitochondrial Permeability Transition Pore; HIF-1: Hypoxic Inducible Factor 1; SIRT1: Sirtuin 1; Nrf2: Nuclear factor (erythroid-derived 2)-like 2.

The mechanisms by which ROS and RNS generation leads to IPC mediated protection are not fully understood, but most likely involves reactive species activating numerous adaptive signaling pathways (Fig. **3**). Redox regulated signaling pathways can occur either by direct conformational change of the protein or through cysteine-rich redox-sensitive proteins, such as that occurs in the proteins thioredoxin and glutathione S-transferase. The reactive species causes disulfide bonds between the cysteine-rich proteins allowing for the formation of dimer and multimers which most likely enhances their cytoprotective activity. Similarly, there are many other molecules that are regulated by ROS and RNS, and are important modulators of the cellular response to ischemia (Table **1**).

### PKC Epsilon

PKC Epsilon (PKCε) is a serine/threonine kinase which belongs to the novel subfamily of PKC isozymes. A central role for PKCε in the IPC neuroprotective response has been demonstrated in both the heart and brain [103, 104]. Inhibition of PKCε, with εV1-2, during and following IPC is sufficient to attenuate IPC neuroprotection against ischemia in several models of cerebral ischemia [104]. Similarly cardiac specific overexpression of PKCε in mice protects against ischemic damage; whereas the IPC neuroprotection is lost in PKCε knockout mice [103]. 

There is increasing evidence that low levels of ROS formation activate PKCε during IPC. A previous study demonstrated that rabbit hearts were protected against ischemia by coronary artery infusion of free radicals which could be blocked by PKC inhibition [39]. Conversely, cardioprotection against ischemia/reperfusion mediated injury, by PKC activation, was not prevented by the ROS scavenger 2-mercaptopropionyl glycine (MPG) suggesting an upstream effect of ROS on PKC activation [105, 106].

The family of PKC isozymes is extremely sensitive to the redox state of the cell. Blocking ROS formation with the free radical scavenger N-acetyl-l-cysteine can impede PKCε translocation to the mitochondria. It has also been demonstrated that photoexcitation-induced ROS formation increased membrane translocation of PKCε. The regulation of PKCε by ROS is likely the result of a unique redox-sensitive domain of the PKC molecule. Oxidation of cysteine-rich motifs on the N-terminal blocked the auto-regulation by PKC thus allowing for activation [107]. The C-terminus catalytic domain contains several reactive cysteines which can be targeted by numerous antioxidants resulting in reduced PKC kinase activity [108]. ROS may also modulate PKC activity by activating proteins upstream of PKC molecules. For example, oxidative stress activates phospholipases A2, D and C that produces the lipid secondary messengers arachidonic acid, phosphatidic acid and DAG. These secondary messengers can then activate PKC [109-111].

Data from Costa *et al.,* [52] suggested that mitochondrial localized PKCε induced mitoK^+^_ATP_ channel opening, resulting in a modest increase in mitochondrial matrix hydrogen peroxide. The resulting increase in oxidative stress promoted further PKCε activation. A similar effect was also observed in the brain; immediately following IPC-induction in hippocampal organotypic slices, our group has demonstrated that PKCε translocated to the mitochondria and phosphorylated the mitoK^+^_ATP_ subunit Kir6.2. In the same study, PKCε was able to prevent mitoK^+^_ATP_ channel opening following IPC exposure suggesting a role for PKCε in the opening of mitoK^+^_ATP_ channels and increasing ROS formation in the brain [44]. 

### SIRT1

SIRT1 is a member of the sirtuin family of Class III NAD^+^ dependent deacetylases [112]. A role for SIRT1 in IPC-induced neuroprotection was demonstrated in our laboratory using both *in vitro* and *in vivo* models of cerebral ischemia. Induction of IPC in organotypic slices increased SIRT1 enzymatic activity [113]. Furthermore, blocking SIRT1 with sirtinol abrogated IPC-induced neuroprotection against oxygen and glucose deprivation (OGD) induced cell death. Similarly, IPC increased SIRT1 enzymatic activity and neuroprotection in an *in vivo* rat model of cardiac arrest [114]. SIRT1 is also activated in the heart following IPC and provides cardioprotection from ischemia and coronary artery occlusion [115-117]. 

Recent research indicates that sirtuin expression and activity can be regulated by oxidative stress; conversely, sirtuins can also regulate ROS concentrations through protein target deacetylation. Oxidative stress can regulate SIRT1 protein activity in several ways: at the transcriptional level; by altering the rate of SIRT1 proteasomal degradation; and by regulating SIRT1 enzymatic activity. At the gene level, mild oxidative stress induced the expression of SIRT1 [118]. However, the transcriptional control program by which this occurs is not fully understood. Interestingly, activation of the hypoxic inducible transcription factor (HIF) can directly activate SIRT1 expression [119]. As will be discussed below, HIF is a target of ROS mediated protein signaling. Therefore ROS may regulate SIRT1 expression indirectly through HIF activation. Recently it has been demonstrated that SIRT1 is covalently modified by enhanced levels of oxidative stress. These chemical modifications reduced SIRT1s enzymatic activity and targeted SIRT1 for proteasomal degradation [120]. Similarly, enzymatic activity of SIRT1 has been shown to be inactivated by posttranslational deSUMOylation during exposure to oxidative stress [121]. 

SIRT1 is known to regulate the expression of antioxidant genes such as sestrins, manganese superoxide dismutase (MnSOD) and glutathione peroxidase 1 [122]. SIRT1 regulates antioxidant levels through the interaction of multiple transcription factors such as p53 and the forkhead box O (FOXO) transcription factor family [123-126]. At low levels of oxidative stress, SIRT1 has been demonstrated to deacetylate and activate FOXO3a which induces the expression of the antioxidant proteins MnSOD and catalase [127, 128]. At higher levels of hydrogen peroxide, however, SIRT1-FOXOX3a induces apoptosis. In the heart, up-regulation of SIRT1 by pressure overload induced oxidative stress activated protective mechanism such as the expression of catalase [129]. Paradoxically, mice expressing high levels of SIRT1 protein demonstrated increased oxidative stress, apoptosis and cardiac hypertrophy. Therefore it has been suggested that sirtuins may act as oxidative stress sensors, activating protective signaling pathways when the oxidative stress is low and activating pro-death signaling pathways when the oxidative stress in high. This fact may account for some contradictory results found in previous studies concerning both pro-survival and pro-death functions of SIRT1. 

### HIF

**Fig. (4):**
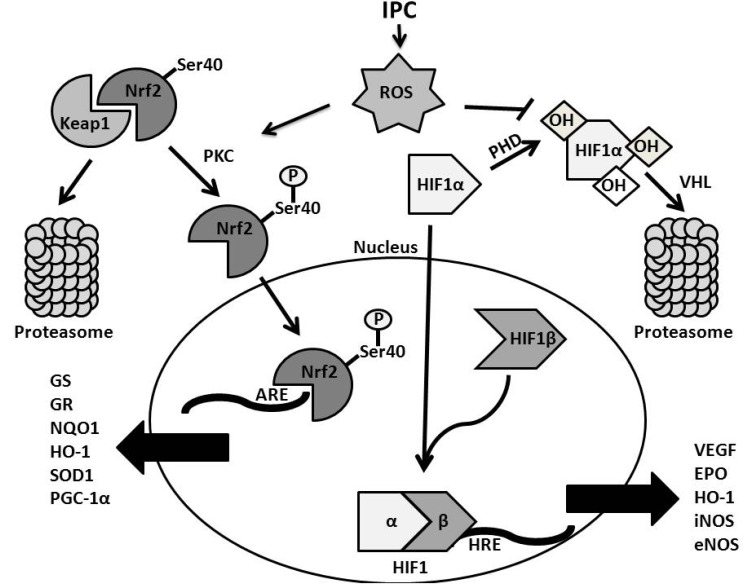
**Summary diagram of Nrf2 and HIF1 stimulation in presence of oxidative stress**. In the presence of ROS, generated by IPC, PKC will become activated to phosphorylate Nrf2 at the Serine 40 residue. This particular residue is the critical site of PKC control, although other kinases can phosphorylate additional sites on Nrf2. Nrf2 will then dissociate from its cytosolic repressor, Keap1, and translocate to the nucleus. Upon binding to ARE, Nrf2 transcribes several genes including those for energy regulation, mitochondria biogenesis, and antioxidant expression. Under normal or high oxygen tension, Nrf2 remains bound to Keap1, and is eventually targeted for ubiquitination and proteasomal degradation. The pathway on the right describes activation of HIF1 in the presence of ROS. The inducible subunit of HIF1, HIF1α, is regulated by PHD. Under normal oxygen levels, PHD will hydroxylate HIF1α. This allows VHL to ubiquitinate HIF1α and target it for proteasomal degradation. However, in the presence of oxidative stress, PHD is inactivated and HIF1α can translocate to the nucleus. In the nucleus, HIF1α binds to the constitutively expressed HIF1β subunit, and together these subunits form the functional HIF1 factor. This factor then binds to HRE on the genome and transcribes several genes involved in hypoxic adaptation, including genes for energy metabolism, angiogenesis, and red blood cell production. Nrf2: Nuclear factor (erythroid-derived 2)-like 2; Keap1: Kelch-like ECH-associated protein 1; PHD: Prolyl hydroxylase; HIF: Hypoxic Inducible Factor; Ser: Serine; OH: Hydroxyl ; VHL: Von Hipple-Lindau; VEGF: Vascular endothelial growth factor; EPO: Erythropoietin; HO-1: Heme Oxygenase 1; iNOS: inducible nitric oxide synthase; eNOS: endothelial-derived nitric oxide synthase; GS: Gluthathione Synthase; GR: Glutathione Reductase; NQO-1: NAD(P)H dehydrogenase [quinone] 1; SOD1: Super Oxide Dismutase 1; PGC-1α: Peroxisome proliferator-activated receptor gamma coactivator 1-alpha.; ARE: Antioxidant Response Element; HRE: Hypoxic Response Element; ROS: Reactive Oxygen Species; PKC: Protein Kinase C.

Hypoxia inducible factors (HIF) are involved in cellular adaptation to hypoxia [130] (Fig. **4**). During low oxygen tensions or hypoxic stress, HIF molecules form heterodimers, consisting of an oxygen-sensitive HIF-1α subunit and a constitutively expressed HIF-1β subunit. Together, these subunits produce active HIF, which acts as a transcriptional factor and binds to hypoxia response elements (HRE) residing in the promoters of HIF target genes. The two predominant isoforms of HIF exist as HIF-1 and HIF-2; together, HIF-1 and HIF-2 are proposed as the main HIF molecules that confer adaptation to hypoxic stress. There exists, however, subtle differences between the two molecules in terms of structure and pathology when single knockouts of the respective HIF isoform are present in mice [131]. Aside from these differences, HIF-1 will be the main focus of this review. Principally, the two categories of HIF-targeted gene expression include genes involved in adaption to hypoxia and enzymes involved in metabolism. HIF activation results in up-regulation of genes to protect a cell from hypoxia, such as vascular endothelial growth factor (angiogenesis) [132, 133], erythropoietin (erythropoiesis) [132], and phosphofructokinase (glycolysis) [134]. 

However, regulation of HIF is under the control of prolyl hydroxylases (PHD) [135]. In normoxia, oxygen activates PHD leading to hydroxylation and inactivation of HIF. The hydroxylation of HIF targets the molecule for ubiquitinated degradation. If oxygen drops to hypoxic conditions, HIF is not hydroxylated but instead heterodimerizes with HIF1 and translocates to the nucleus, where it induces gene transcription. In addition, HIF can also instigate mitochondrial autophagy through the expression of an autophagy stimulating protein, BNIP3 (Bcl-2/adenovirus E1B 19 kDa interacting protein), thereby preventing mitochondrial release of cytochrome *c* and attenuating mitochondrial ROS production [136]. Isoforms of PHDs, such as PHD2/PHD3 are also located on HRE [137]; thus even in the absence of oxygen, prolonged HIF-1 activation can cause a negative feedback loop, eventually leading to the degradation and suppression of the HIF signal. Further transcriptional regulation of HIF is under control of Factor Inhibiting HIF-1 (FIH-1) [138]. Under oxygenated conditions, there are asparagine residues on HIF that are hydroxylated by FIH-1 [139]. This hydroxylation prevents binding of p300, a coactivator of HIF, to HIF’s COOH-terminal transactivation domain (CTAD) [140, 138]. The CTAD domain is also sensitive to hypoxic stress, as low oxygen tensions inhibit the hydroxylating activity of FIH-1 [139, 138]. These coactivators are pivotal in regulating transcriptional activity of HIF-1 during hypoxia.

#### HIF-1 Regulation Through Reactive Oxygen Species

As previously discussed, the ETC of the mitochondria is one of the principal producers of ROS inside the cell. However, HIF-1 may bind to one of the 5 activation sites on Lon, a mitochondrial protease which degrades the cytochrome c oxidase (COXIV) subunit COXIV-1 and allows HIF-1 mediated increases in COXIV-2 [141]. The advantage of COXIV-2 upregulation is that less ROS is generated during cellular respiration, while less oxygen is consumed. By reducing the ratio of ROS production to oxygen consumed, efficiency of cellular respiration is increased under situations when environmental oxygen is at a premium. In addition, redistribution of oxygen from the mitochondria to non-mitochondrial cellular compartments may also decrease activation of PHD in order to increase HIF activation [141]. A previous study demonstrated that under hypoxic conditions, ROS generated from complex III of the ETC increased accumulation of HIF-1*α *through oxidizing the ferrous state of the dioxygenase iron cofactor necessary for proper PHD functioning [142]. Absence of mitochondrial DNA from yeast, which precluded formation of an electron transport chain, resulted in reduced free radical species generation and attenuation of HIF-targeted gene transcription. The results of the above studies suggest ROS is an important signaling mechanism for HIF-dependent cellular adaptation to hypoxia. 

#### HIF-1 Interaction with Nitric Oxide 

In addition, nitric oxide has been shown to modulate HIF activity [143, 144]. The use of NO donors demonstrated an increase in HIF stabilization and target gene transcription under non-hypoxic conditions [145-147]. However, another study demonstrated that nitric oxide was shown to decrease HIF-1*α *stabilization in bovine pulmonary artery endothelial cells under hypoxic conditions through a cGMP mediated mechanism [148]. Therefore, nitrosylation rather than cGMP activation is ascribed to be the mechanism by which nitric oxide mediates hypoxic adaptation through HIF-1. HIF-1 was also observed to be nitrosylated on select cysteine residues, which resulted in increased activation [149, 150]. In HEK-293 cells that expressed human iNOS, studies showed that high concentrations of NO induced HIF stability regardless of oxygen concentration, but low NO concentrations promoted HIF degradation [151]. Conversely, a previous study demonstrated that low NO levels may redistribute oxygen by inhibition of cytochrome *c* oxidase through high-affinity binding; this action displaces oxygen, and decrease mitochondrial respiration and subsequent oxygen consumption. These effects resulted in increased oxygen levels in other cellular compartments, allowing oxygen to inhibit PHD [152]. Thus, there exists a critical balance between activation and inhibition of HIF-1 through nitric oxide, and future studies must investigate the delicate mechanism that governs this phenomenon.

#### Activation of HIF-1 During Induction of IPC

If hypoxic stress activates HIF, then transient, preemptive ischemic insults may also activate HIF without producing injury to cells. Indeed, induction of IPC increased the activation of HIF-1α in various IPC-induced tissue models. In the newborn piglet brain, IPC treatment increased mRNA expression of HIF-1α while also decreasing cell death in the hippocampus and cortex following a 30 minute lethal ischemic insult [153]. IPC-treated primary rat astrocyte cultures showed decreased cell death following oxygen-glucose deprivation; the IPC-treated cultures demonstrated increased nuclear accumulation of HIF-1α as well as increased levels of proteins under HIF-1α transcriptional control. In another study, pharmacologic preconditioning of HIF-1 through known HIF-1 activators cobalt chloride (CoCl_2_) and desferrioxamine (DFX) protected neonatal rat brains from lethal ischemia. The neuroprotection observed was approximately the same as that achieved through IPC treatment. IPC treatment also increased mRNA of HIF-1 α and HIF-1β [154]. These studies suggest that IPC-mediated protection is due, at least in part, through HIF-1 mediated pathways. 

### Nrf2

An important transcription factor which is activated in the presence of free radicals and electrophilic stress is nuclear factor erythoid-2 related factor (Nrf2) (Fig. **4**). Nrf2 is involved in protecting the cell from the damaging effects of oxidative stress by binding to antioxidant response elements (ARE), located in the regulatory domains of its target genes [155]. Under basal conditions of oxygen tension, there exists a cytosolic protein, known as Keap1 (Kelch-like ECH-associated protein 1), that functions to down regulate the activity of Nrf2 [156]. Keap1 complexes with Nrf2 and activates Nrf2 ubiquitination, eventually leading to its degradation under normal conditions. However, under certain conditions, Keap1 or Nrf2 may be chemically modified through phosphorylation [84, 157], deacetylation [158], and S-nitrosylation. These chemical modifications enhance Nrf2 disassociation from Keap-1, thus facilitating Nrf2 nuclear translocation and subsequent Nrf2-dependent gene expression. Upon nuclear translocation, Nrf2 binds to ARE in the regulatory domain of its target genes; examples of antioxidant genes under Nrf2 transcriptional control include glutathione synthase [159], heme oxygenase-1 [160], and catalase [161, 162].

ROS has been suggested to regulate activation of Nrf2 following ischemia through kinase activation. Subsequent phosphorylation of Nrf2 enhances Nrf2 dissociation from Keap1 and allows Nrf2 to express antioxidant enzymes and other proteins to better adapt the cell to oxidative stress [163]. Nrf2 has a ubiquitous expression, as Nrf2 has been shown to induce antioxidant gene transcription in rat liver [164], lung [165], brain [166] and heart tissue [161]. Nrf2 was shown to be up-regulated following a 50% reduction in cerebral blood flow in mice; the resulting cerebral oligemia in mice led to increased oxidative stress and subsequent activation of Nrf2 in neurons predominantly in cerebellar Purkinje cells and cingulate cortex [167]. 

In addition to ROS, NO has been shown to activate Nrf2 through S-nitrosylation of cysteine residues residing on Keap1 in cultured rat pheochromocytoma cells. This chemical modification allowed Keap1 to dissociate from Nrf2, allowing Nrf2 to translocate from the cytosol to the nucleus. In addition to S-nitrosylation, this study suggested that nitric oxide could activate PKC-dependent phosphorylation and subsequent dissociation of Nrf2 from Keap1 [168]. A recent study has also suggested that SIRT1 was inhibitory on Nrf2’s transcriptional activity [158]. More importantly, this study also suggested a novel regulation of Nrf2 after separation from Keap1, such that acetylation of the dissociated and nuclear-translocated Nrf2 enhanced its binding to ARE. However, conflicting results have been reported with the use of resveratrol, a polyphenolic antioxidant known to activate SIRT1. Resveratrol was demonstrated to stabilize and restore levels of Nrf2 in the cerebellum in a rodent model of fetal alcohol syndrome [169]. Yet another study suggested that the use of histone deacetylase inhibitors increased Nrf2 activation following focal cerebral ischemia in mice, and resulted in decreased infarct volumes when administered shortly after induction of focal cerebral ischemia [166]. This last study suggests that inhibition of SIRT1 and other sirtuin enzymes may activate Nrf2, conferring neuroprotection to ischemia. 

There has been extensive debate as to whether transient hypoxic stress activates Nrf2 protective pathways. A previous study demonstrated upregulation of Nrf2-targeted gene transcription following IPC in human and rat astrocytes; more importantly, the observed decrease in cell death due to induction of IPC was abrogated in homozygous Nrf2 knockout rats, suggesting that Nrf2 could mediate an important role in IPC mediated neuroprotection [170]. The group *Bell et al., *demonstrated that pure neuronal cultures were unable to upregulate antioxidant genes under Nrf2-transcriptional control, suggesting that astrocytes are the primary source of Nrf2 production and activation. Finally, homozygous Nrf2 knockout mice were not protected from induction of IPC or exposure hydrogen peroxide following OGD, supporting the contributive role of Nrf2 to mediating IPC-induced neuroprotection [171]. 

## CONCLUSIONS

Therapeutic intervention for the treatment of cerebral ischemia has proven extremely elusive and has ended in the failure of numerous clinical trials. However the brain is endowed with an innate neuroprotective program against ischemic damage which is activated by mild ischemic exposure. The therapeutic potential of IPC has increased interest in understanding the signaling pathways and mechanism by which IPC mediates neuroprotection. As discussed in this review, the level of ROS and RNS generation is thought to be a pivotal trigger in activating either survival or death pathways in the cell following ischemia. It is also becoming clear that IPC induced ROS formation may serve as the trigger for activation of IPC mediated neuroprotective pathways. 

The ultimate goal and clinical application of IPC research is to gain pharmacological access to this neuroprotective state in individuals undergoing procedures in which necessary periods of ischemia are provoked in healthy tissue and in individuals at high risk of stroke. The use of preconditioning in the clinic has hitherto been primarily conduced in the heart during cardiac bypass graft (CABG) surgery and angioplasty. For example Yellon *et al*., [172] induced IPC by aortic cross-clamping for 2 min followed by 3 min of reperfusion two times prior to CABG surgery. Patients which received IPC treatment in this study maintained ATP levels in ventricular biopsies and displayed reduced serum troponin T levels. The accumulated results of 22 such studies were summarized in a meta-analysis which demonstrated that IPC was associated with reduced ventricular arrhythmias, reduced inotrope requirements, and decreased intensive care stay [173].

The feasibility of pharmacological conditioning is illustrated by the use of Nicorandil, a mitoK^+^_ATP_ channel opener, in acute intervention for myocardial infarction. Treating patients with Nicorandil prior to angioplasty treatment displayed reduced left ventricular remodeling and improved ventricular function [174]. However, clinical proof of concept application of conditioning mimetics has been troublesome and has met with numerous clinical trail failures (Reviewed in Hausenloy and Yellon [175]). The preclinical application of other conditioning mimetics has yielded very promising results. For example, numerous animal studies have demonstrated the protective effects of resveratrol, a SIRT1 activator, desferrioxamine, a HIF-1 activator, and εψRACK, a PKCε activator, against ischemic damage [176-181, 114, 113, 182-188]. 

The comorbidities of patients have complicated the bench to bedside transition of preconditioning. Although many proteins and molecular pathways have been implicated in IPC-mediated neuroprotection and oxidative stress signaling, there is still much to be learned about the protective effects of IPC against ischemic injury and the role of ROS and RNS in this process.

## Figures and Tables

**Table 1.** Mechanisms of ROS Induced Cellular Damage and Protection

Excessive ROS Formation	Result
Inactivation of electron transport chain proteins	Reduced ATP production
Inactivation of prostacyclin (PGI_2_) synthase	Reduced prostacyclin/ increased inflammation
Lipid peroxidation	Membrane damage/ inflammation
Protein oxidation	Alteration in protein function
DNA damage	Activation of PARP and apoptosis
Delta PKC activation	Mitochondrial dysfunction/apoptosis
**Mild ROS Formation**	**Result**
PKCε activation	Activation of survival MAPK proteins
SIRT1 activation	Increased expression of the antioxidant enzymes MNSOD and glutathione
Nrf-2 nuclear translocation	Increased expression of the antioxidant enzymes glutathione synthase, heme oxygenase and catalase
HIF-1 stabilization and activation	Expression of angiogenic proteins, erythropoietin, phosphofructokinase, increased expression of COXIV-2 (which reduces ROS formation by the electron transport chain)
